# Sandwich method in Crowe type 4 hip arthroplasty surgery: a retrospective study on a novel technique for osteotomy line union

**DOI:** 10.1097/MD.0000000000042784

**Published:** 2025-06-13

**Authors:** Mehmet Coşkun, Abdurrahman Aydin, Deniz Akbulut

**Affiliations:** a Van Akdamar Hospital, Van, Turkey; b Düzce Akcakoca State Hospital, Düzce, Turkey.

**Keywords:** Crowe type 4, developmental dysplasia of hip, osteotomy, sandwich method

## Abstract

In hip arthroplasties performed on patients with Crowe type 4 dysplasia, femoral shortening is often required to position the hip into the true acetabulum. Nonunion at the osteotomy site is one of the significant comorbidities in osteotomies that lower the hip into the true acetabulum. This study aimed to evaluate the clinical and radiological outcomes of hip arthroplasty with femoral shortening in patients with Crowe type 4 dysplasia using the technique we developed. This study retrospectively evaluated the outcomes of 96 (117 hips) patients who underwent subtrochanteric transverse shortening osteotomy due to Crowe type 4 dysplasia between January 2016 and December 2020, with a minimum follow-up period of 24 months. Harris Hip Score (HHS) was used for clinical evaluation of the patients, followed by comparison of preoperative and postoperative values. Radiological evaluation included assessments of union time, leg length discrepancy, and union rates. Intraoperative and postoperative complications were noted. The HHS had a mean preoperative value of 54.66 ± 7.47 and increased significantly postoperatively to a mean value of 93.82 ± 7.00 (*P* < .05). Changes in HHS (~39.2) surpass the minimal clinically important difference threshold. At the final follow-up, the union was observed in 115 (98.3%) hips. Despite a follow-up period exceeding 24 months, nonunion was radiologically observed in 2 (1.7%) hips. Our observations had relatively lower rates than those reported in literature. The preoperative and postoperative leg length discrepancies were measured as 5.03 ± 1.09 cm and 0.64 ± 0.74 cm, respectively (*P* < .05). Intraoperatively, femoral fractures were observed in 12 (10.3%) hips, dislocations in 5 (4.3%) hips, and sciatic nerve injury in 4 (3.4%) hips. In hip arthroplasties performed on patients with Crowe type 4 dysplasia, supporting the strut graft around the subtrochanteric transverse osteotomy site with cancellous bone taken from the trochanter major and femoral head reduces the risk of nonunion, and satisfactory clinical outcomes can be achieved with this method.

## 1. Introduction

Developmental dysplasia of the hip (DDH) is a prevalent health concern in developing countries, negatively affecting the quality of life of patients and leading to serious health problems.^[1]^ It presents with symptoms, such as hip pain, limited mobility, and difficulties in daily activities.^[2]^ The treatment of Crowe type 4 DDH in adults requires highly complex techniques.^[1]^ Crowe type 4, in particular, makes surgical interventions like total hip arthroplasty (THA) exceedingly challenging. Advanced surgical techniques, for example, subtrochanteric shortening osteotomy, are often necessary.^[1]^ Examining such cases and evaluating the efficacy of treatment methods are crucial for improving surgical outcomes and reducing complication rates.^[3]^ For patients who develop secondary osteoarthritis due to hip dysplasia and are unresponsive to conservative treatments, hip arthroplasty is the ideal treatment. Performing THA in patients with Crowe type 4 DDH presents surgical difficulties. Despite the challenges, acetabular reconstruction in the true acetabulum is the gold standard as it helps prevent loosening (wear), limping, and impingement. Following acetabular reconstruction in the true position, femoral shortening osteotomy is often necessary to balance leg length and avoid nerve damage caused by excessive lengthening. Investigating the outcomes of various treatment methods for Crowe type 4 dysplastic hip arthroplasties, a complex case group, is essential for contributing to the advancement of surgical techniques.^[4]^

Although many different shortening osteotomy techniques have been described (e.g., step-cut, oblique, adapting the trochanter major to the prosthesis, and distal shortening), subtrochanteric shortening osteotomy is the most commonly used method. Among subtrochanteric osteotomies (step-cut, oblique, transverse, and chevron), the transverse osteotomy is the most frequently employed. In these techniques, ease of application and derotation are the most important advantages. However, the major disadvantage are the mismatch between the proximal and distal bone surface areas and smaller fusion surface than other osteotomy techniques, which makes bone union more challenging. Because it is not possible to interlock the proximal and distal parts, this technique has weak rotational stability. In the center where this osteotomy technique is applied, a strut graft is wrapped around the osteotomy site with a cable to enhance stability.^[3]^

Several studies have been conducted on THA and subtrochanteric transverse shortening osteotomy in patients with Crowe type 4 hip dysplasia. These show the effect of femoral stem type and osteotomy type on union at the osteotomy line, whereas none have used a spongiosis graft from the trochanter major and femoral head at the osteotomy line for nonunion, which was done in this study. However, more data are required, particularly regarding surgical outcomes and complication rates in these patients.^[1,5,6]^ The present study aimed to assess the surgical outcomes of THA and subtrochanteric transverse shortening osteotomy in patients with Crowe type 4 DDH to demonstrate the efficacy and reliability of these techniques. By examining long-term outcomes and complication rates, we seek to provide more comprehensive information on the effectiveness of these surgical methods.^[2]^

Our goal is to show that adding impaction grafting with cancellous grafts taken from the trochanter major and femoral head to subtrochanteric transverse shortening osteotomy can reduce the risk of nonunion. To determine the efficacy and reliability of these techniques and to analyze complication rates, we evaluated the surgical outcomes of THA and transverse subtrochanteric shortening osteotomy in patients with Crowe type 4 DDH.

The primary hypothesis is that the surgical outcomes of THA and subtrochanteric transverse shortening osteotomy in patients with Crowe type 4 DDH will demonstrate the efficacy and reliability of these advanced techniques. Supporting hypotheses are that these surgical methods will improve functional outcomes, reduce complication rates, and improve quality of life.

## 2. Materials and methods

**Figure 1.**
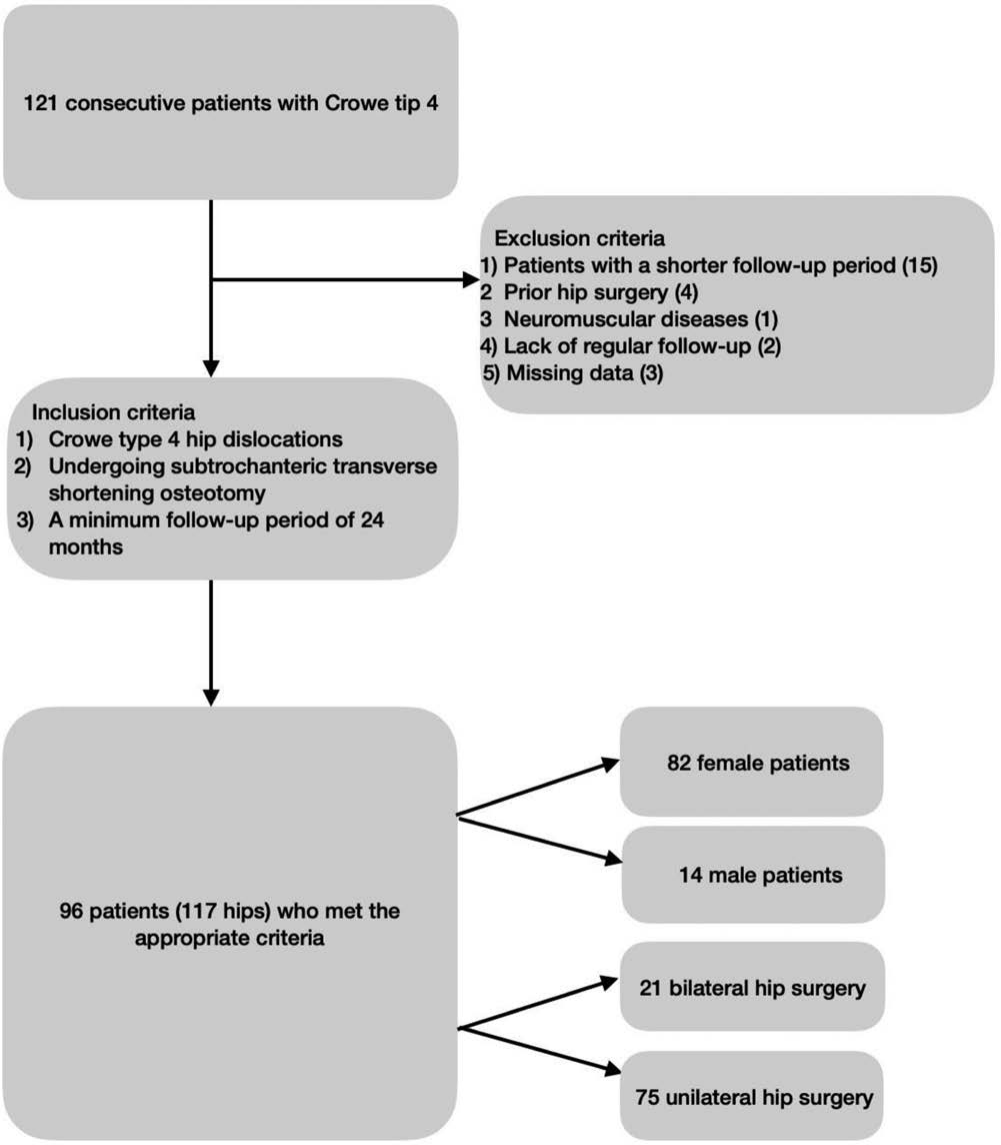
Flowchart of the study.

The outcomes of 96 (117 hips) patients who underwent THA and subtrochanteric transverse shortening osteotomy due to Crowe type 4 DDH between January 2016 and December 2020 were evaluated. The inclusion criteria for the study were the presence of Crowe type 4 hip dislocation, undergoing subtrochanteric transverse shortening osteotomy, and a minimum follow-up period of 24 months. The exclusion criteria were a shorter follow-up period (15 patients), prior hip surgery (4 patients), neuromuscular disease (1 patient), lack of regular follow-up (2 patients), and missing data (3 patients; (Fig. 1).

### 2.1. Surgical technique

**Figure 2.**
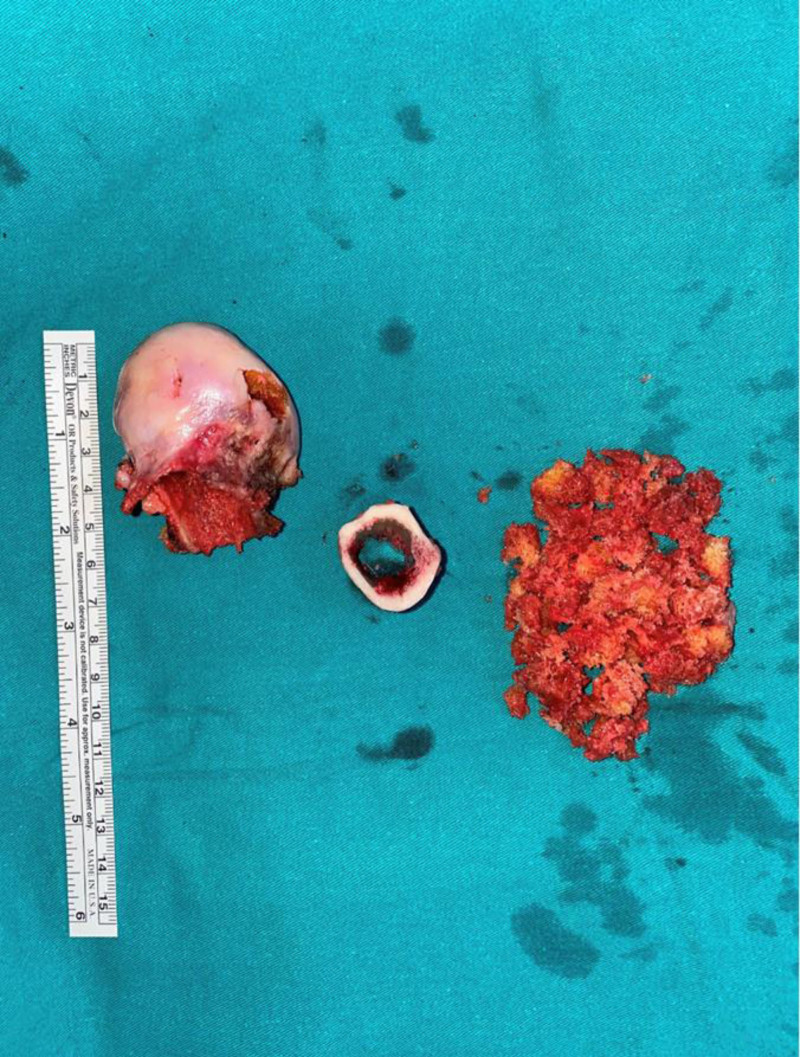
The removed femoral head, cortical bone extracted from the shortening site, cancellous graft taken from the femoral head, and greater trochanter.

**Figure 3.**
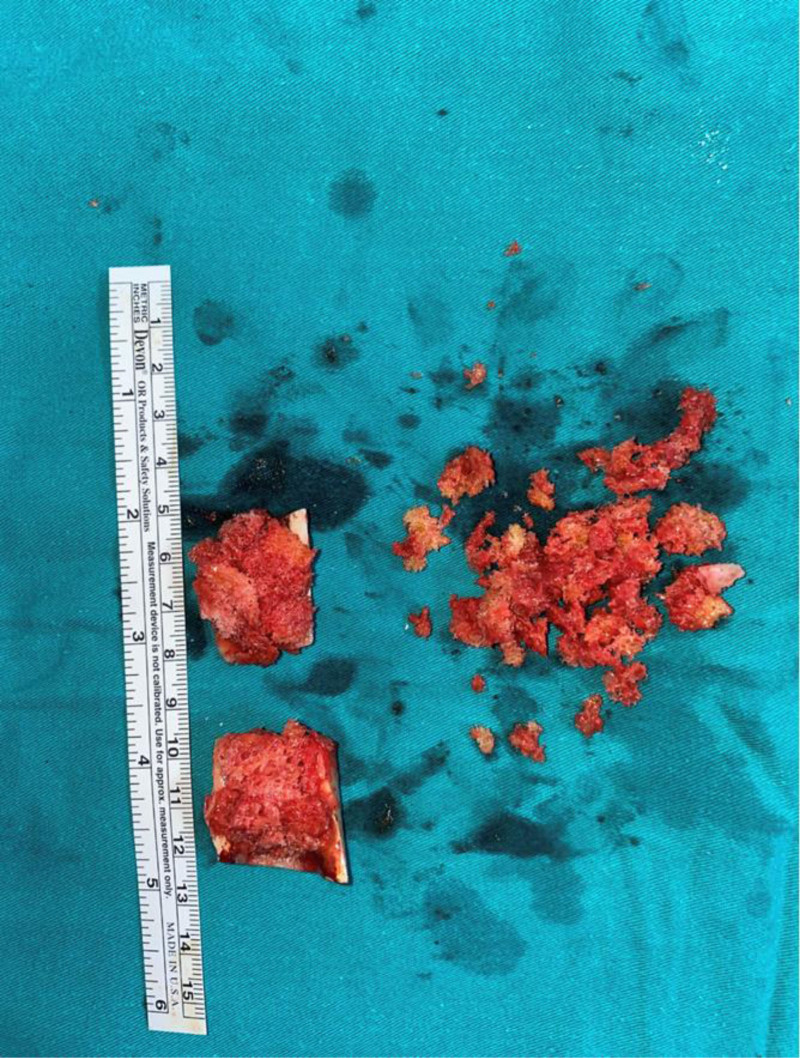
After the extracted cancellous graft, the prepared state is impacted and placed inside the strut graft. Spongios bone graft removed from the femoral head and greater trochanter is prepared to be sandwich in the struct graft.

**Figure 4.**
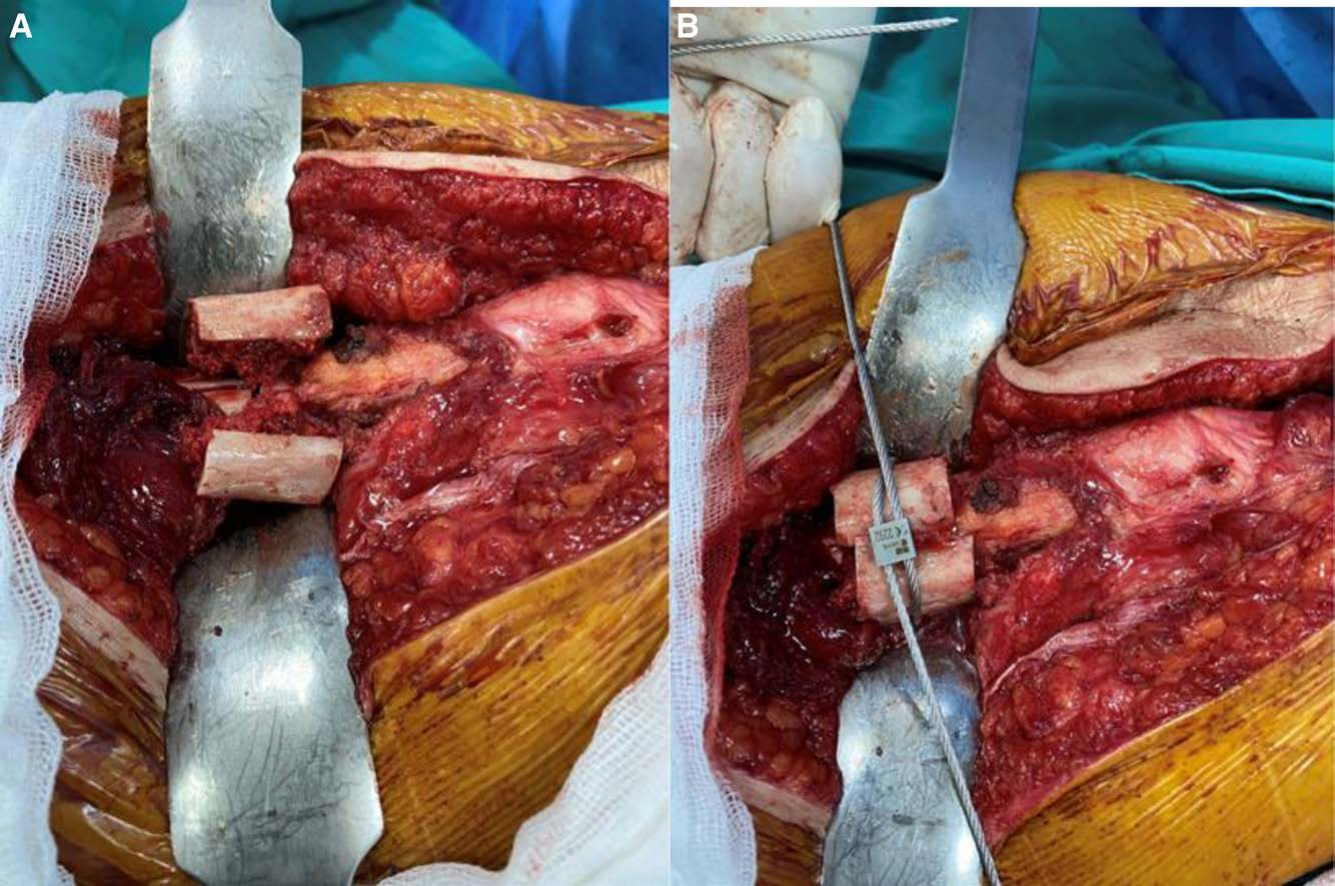
(A) Placement of the strut grafts, prepared by impacting the cancellous graft, at the osteotomy site. The graft, which was prepared with an average length of 2 cm, was placed on the osteotomy line to cover the 2 edges of the sandwich. A sandwich-shaped graft with 2 closed edges was placed centrally on the osteotomy line. (A) Fixation of the placed strut grafts with a cable.

The surgical procedure was performed under general/spinal anesthesia by 2 experienced arthroplasty surgeons, with the patient in the lateral decubitus position using a posterolateral approach. In all cases, a Wagner Cone™ stem (Zimmer, Warsaw) and a Zimmer acetabular cup were used. A femoral neck incision was performed following the standard posterolateral approach for hip arthroplasty. During femoral reaming, cancellous grafts from the trochanter major, the medullary canal, and the femoral head were harvested and preserved (Fig. 2). A subtrochanteric transverse osteotomy was performed. After accessing the true acetabulum, acetabular reconstruction was completed. Once the amount of shortening was determined, the resected femoral segment was prepared as a strut graft. After the original stem was impacted, the osteotomy site was filled with the preserved cancellous grafts. The cancellous grafts were impacted in a sandwich configuration within the strut graft (Fig. 3). The strut graft was adjusted to an average length of 2 cm, with the spongiosa graft sandwiched in between, and the graft was divided into 2 equal parts to align the osteotomy line longitudinally. The strut graft and the impacted cancellous grafts were wrapped with a cable (Titanium and cobalt chromium alloy, 1.8 mm in diameter, and 2 1.5-mm braided wire cerclages wrapped once around the bone and closed with a crimp) to ensure rotational stability (Fig. 4A and 4B). In cases of incompatibility between the medullary canal widths of the proximal and distal parts after osteotomy, the size of the femoral stem was increased. However, this increased the possibility of femoral fissure-fracture. In case of fracture of the trochanter and distal femur or in cases with insufficient rotational stability of the prosthesis, additional plating may be required. In such instances, the strut grafts should not be placed fully laterally to avoid interfering with the plate.

### 2.2. Postoperative protocol

All patients received prophylactic antibiotics, analgesics for pain relief, and anticoagulant prophylaxis for deep vein thrombosis. Patients undergoing surgery were given partial weight-bearing with toe contact for 6 weeks after which complete weight-bearing was given. Radiographs were taken monthly until the osteotomies healed, the point at which the patients could bear full weight.

### 2.3. Data evaluation

The mean follow-up period was 50.34 months (range, 26–98). Clinical evaluation was performed using the Harris Hip Score (HHS), and preoperative and postoperative values were recorded. Radiologically, measurements of leg length discrepancy (LLD) were conducted. Bone union times and rates were assessed. The complications were documented in detail.

Concerning the HHS, the minimal clinically important difference (MCID) of 8 was employed for comparing preoperative and postoperative values.^[7]^

### 2.4. Data analyses

The statistical analysis was carried out using SPSS 26.0 software (SPSS Inc., IBM, New York). The numerical and categorical variables were presented as means and standard deviations and frequencies and percentages, respectively. The nonparametric Wilcoxon signed-rank test was used to compare preoperative and postoperative measurements. A *P* value < 0.05 was considered statistically significant in all analyses.

The study protocol was approved by the University of Health Sciences Van Training and Research Hospital ethical committee institutional review board no. 2022/10-04, May 11, 2022), and all patients provided written informed consent.

## 3. Results

The mean age of the 96 (117 hips; 82 [85%] women; 14 [15%] men) patients included in the study was 30.74 ± 10.57 years. Bilateral surgery in different sessions was performed in 21 (21.9%) patients. The mean follow-up period was 50.34 ± 10.98 months (range, 26–98). The demographic characteristics of the patients are presented in detail in Table 1.

**Table 1:** Demographic characteristics of the patients.

Characteristic	Value
No. of patients	96
No. of Hips	117
Mean age (yr)	30.74 ± 10.57
Gender (female/male)	82/14
Mean body mass index (kg/m^2^)	22.2 ± 3.1
Bilateral surgery	21 patients
Side (right/left)	59/58
Mean follow-up duration (mo)	50.34 ± 10.98

During surgery, the mean amount of femoral shortening was 3.46 ± 0.85 cm (range, 3–5.5). The mean operation time was 139.7 ± 40.3 minutes (range, 95–196).

When clinical outcomes were evaluated, the preoperative and postoperative HHSs were 54.66 ± 7.47 and 93.82 ± 7.00, respectively. In addition, the preoperative and postoperative Western Ontario and McMaster University Osteoarthritis Index scores were 48.5 ± 21.8 and 18.0 ± 11.8, respectively. The preoperative and postoperative LLDs were 5.03 ± 1.09 and 0.64 ± 0.74 cm, respectively (Table 2). Changes in the HHS (~39.2) surpass the MCID threshold.

**Table 2:** Clinical and radiological outcomes.

Measurement	Preoperative	Postoperative
Harris Hip Score	54.66 ± 7.47	93.82 ± 7.00
Western Ontario and McMaster University Osteoarthritis Index Score	48.5 ± 21.8	18.0 ± 11.8
Mean Leg Length Discrepancy (cm)	5.03 ± 1.09	0.64 ± 0.74

**Figure 5.**
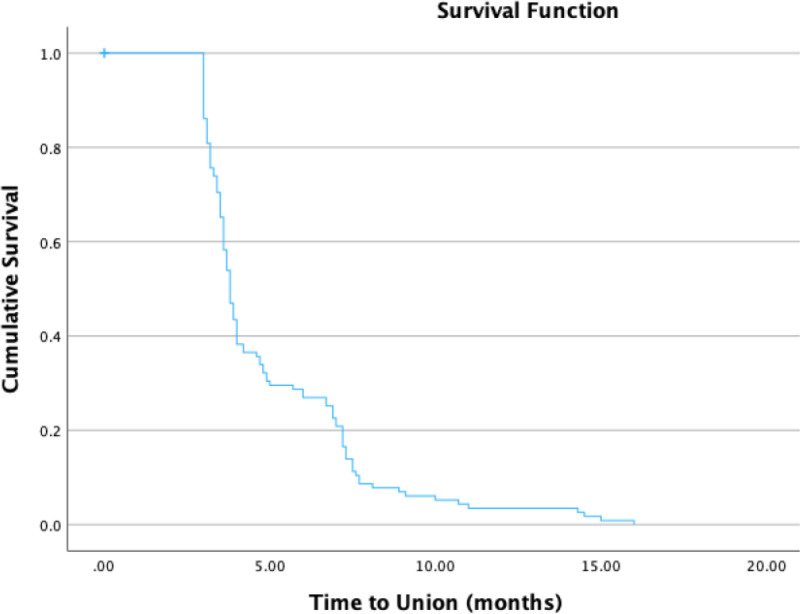
Kaplan–Meier survival analysis curve for union rate/time-to-union.

In patients who underwent subtrochanteric transverse shortening osteotomy, union rates were 61% at 4 months, 90% at 8 months, and 95% at 12 months. The union rate at the final follow-up was 98.3% (115/117 hips), and the mean union time was 6.0 ± 3.0 months (Table 3; Fig. 5). Despite a follow-up period exceeding 24 months, nonunion was radiologically observed in 2 (1.7%) hips. The observed union rates were relatively lower that the rates reported in literature.

**Table 3:** Union rates and times.

Time	Union rate
4th mo	61%
8th mo	90%
12th mo	95%
Union rate at final follow-up	115/117 (98.3%)
Average union time	6.0 ± 3.0 months

The most common complication was femur fracture in 12 (10.3%) hips, followed by dislocation in 5 (4.3%) hips, sciatic nerve injury in 4 (3.4%) hips, and nonunion at the osteotomy site in 2 (1.7%) hips (Table 4).

**Table 4:** Complications.

Complication	n (%)
Intraoperative femur fracture	12 (10.3)
Hip dislocation	5 (4.3)
Sciatic nerve injury	4 (3.4)
Nonunion at the osteotomy site	2 (1.7)

## 4. Discussion

The results obtained in the present study demonstrated that supporting the strut graft around the subtrochanteric transverse osteotomy site with cancellous bone taken from the greater trochanter and femoral head reduced the risk of nonunion in patients with Crowe type 4 hip dysplasia. In these patients, femoral shortening osteotomy is required in many cases to position the acetabulum in its true center to reduce muscle tension, balance leg length, and prevent nerve damage. In an average follow-up period of 50.34 ± 10.98 months, a statistically significant improvement was observed in HHSs, increasing from 54.66 ± 7.47 preoperatively to 93.82 ± 7.00 postoperatively. Changes in HHS (~39.2) surpass the MCID threshold.

Nonunion was observed in only 2 (1.7%) of the 117 hips operated on in 96 patients. These results demonstrate the effectiveness of the surgical technique used. Particularly, we believe that the sandwich and grafting methods used in the osteotomy site played a critical role in reducing the risk of nonunion. Although various femoral shortening osteotomy techniques have been described, the subtrochanteric transverse shortening osteotomy is widely used owing to its easy application, reduced surgical time, and high range of derotation during surgery. These findings provide a new solution to the challenges of THA in patients with highly dislocated hips and significantly contribute to the literature.

One of the most important findings of the present study was the low nonunion rate of 1.7%. This rate is quite successful compared with the reported rates in literature, which range from 2 to 12.5%.^[8,9]^ Unlike the present study, Kayaalp et al^[3]^ performed shortening osteotomy using Zweymuller stem without additional fixation and reported a similar nonunion rate (1/46, 2.2%). The author emphasized that the most effective factors in terms of union were the proper alignment of the stem and the use of the thickest possible stem considering that graft use was not obligatory. The nonunion rate observed in the present study was lower than the 4.3% delayed union rate reported in the study by Bernasek et al^[10]^ and the 6% nonunion rate reported in the study by Sun et al.^[11]^ We attribute the successful outcome to the sandwich and impaction grafting methods we applied. Nonunion at the femoral shortening osteotomy site is one of the most important complications. Understanding the possible causes of nonunion and exercising care using surgical techniques is crucial to prevent nonunion. During surgery, issues, such as periosteal stripping, thermal damage caused by the osteotomy, and insufficient stability of the femoral stem, can be mitigated with careful attention.

The significant increase in the HHS from 54.66 ± 7.47 to 93.82 ± 7.00 indicates a marked improvement in the functional outcomes of the patients. This result is comparable to, even slightly better than, the increase reported by Wang et al., which went from a preoperative score of 38.8 to a postoperative score of 86.1.^[12]^ Similarly, Mu et al^[1]^ observed a comparable increase from 35.6 to 82.9 in their study. This notable improvement in the HHS demonstrates the effectiveness of the surgical technique and the enhancement in the patients’ quality of life. Concerning the HHS, the MCID was employed for comparing preoperative and postoperative values. Changes in the HHS (~39.2) surpass the MCID threshold.

The average shortening amount of 3.46 ± 0.85 cm is also consistent with the values reported in the literature. For instance, Krych et al.^[13]^ reported an average shortening of 31 mm, whereas Mu et al^[1]^ reported an average shortening of 35 mm. This shortening helps reduce the risk of nerve damage due to excessive elongation while ensuring adequate leg length equality. In a study aimed at determining the osteotomy level for increasing surface area in a subtrochanteric transverse osteotomy, no significant difference was found in the union surface area between osteotomies performed from 0.5 to 2.5 cm distal to the lesser trochanter.^[14]^ Therefore, subtrochanteric osteotomies were routinely performed not at a specific level but at the area where the medulla begins to narrow. In unilateral cases, the decrease in the average clinical LLD from preoperative values to postoperative values of 11.4 mm indicates the effectiveness of this technique. This result is consistent with the mean postoperative LLD of 9 mm reported by Lai et al.^[15]^

The subtrochanteric transverse shortening osteotomy used in the present study had several significant advantages compared with techniques like step-cut, oblique, or chevron osteotomies.^[9,16]^ This technique is easier to implement and reduces the operation time. In addition, it allows for the desired amount of derotation during surgery, providing flexibility in adjusting femoral anteversion.^[13]^ However, one of the main disadvantages of transverse osteotomy is the mismatch of proximal and distal bone surface areas, which offers less union surface than other techniques.^[12]^ To address this disadvantage, we developed a sandwich method that enhances the potential for union through the impaction grafting method at the osteotomy site. In literature, the use of additional fixation methods (plate, cable, and strut grafts) at the shortening osteotomy site is commonly reported.^[17,18]^ However, our technique can achieve sufficient stability through the appropriate stem size and placement. This approach shortens the operation time and reduces potential complications.

The most important complications encountered in the present study were femoral fracture (10.3%), prosthetic dislocation (4.3%), and nerve damage (3.4%). The rate of femoral fractures in the present study was relatively higher than the reported rates in the literature, ranging from 5.2 to 28%.^[1,12]^ This higher rate may be associated with our strategy of upsizing the stem in cases of mismatch between the proximal and distal medullary widths. The prosthetic dislocation rate was consistent with the literature, where Wang et al^[12]^ reported a dislocation rate of 3.9%, and Ollivier et al^[18]^ reported a rate of 10.7%. The rate of nerve damage in the present study was at the lower end of the range reported in the literature, which varies from 5 to 11.3%.^[12,19,20]^ Preventive measures can be taken to reduce the occurrence of these complications, including prophylactic cable application, particularly in narrow femoral canals, to reduce the risk of periprosthetic fractures; paying close attention to soft tissue tension and component positioning to prevent prosthetic dislocation; and avoiding excessive limb lengthening to minimize nerve damage and performing additional shortening if necessary. In addition, intraoperative neuromonitoring could be considered, which would help reduce complication rates and improve patient outcomes.

The limitations include the retrospective design, the fact that our patient population consists of young individuals without additional morbidities and the relatively short follow-up period. We believe that the number of patients excluded because of the short follow-up period does not affect our results owing to their small proportion (12%) in the study population; however, we consider this a limitation. These limitations hinder our ability to assess whether our results apply to broader and more diverse patient groups. To strengthen the validity and generalizability of our findings, future prospective studies with larger, more diverse patient populations and extended follow-up durations are planned to further evaluate the clinical efficacy, safety, and long-term outcomes of the sandwich technique in Crowe type 4 hip dysplasia.

## 5. Conclusion

The present study has demonstrated that our developed technique provides effective and reliable union at the osteotomy site in patients with Crowe type 4 hip dysplasia. Particularly, the applied sandwich and impaction grafting methods contributed to low nonunion rates and high functional outcomes with HHS change above the threshold for MCID. Thus, we recommend this technique in clinical practice, especially for young and active patients. In such surgeries, it should be considered that sufficient stability can be achieved without the need for additional fixation methods at the osteotomy site. However, adapting the technique according to each patients’ anatomical characteristics and surgical challenges is important.

## Author contributions

**Conceptualization:** Mehmet Coşkun, Abdurrahman Aydin.

**Funding acquisition:** Mehmet Coşkun.

**Investigation:** Mehmet Coşkun, Deniz Akbulut.

**Writing—original draft:** Mehmet Coşkun, Abdurrahman Aydin.

**Writing—review & editing:** Mehmet Coşkun, Abdurrahman Aydin.

**Data curation:** Abdurrahman Aydin.

**Formal analysis:** Abdurrahman Aydin.

**Methodology:** Abdurrahman Aydin, Deniz Akbulut.

**Project administration:** Abdurrahman Aydin, Deniz Akbulut.

**Supervision:** Abdurrahman Aydin, Deniz Akbulut.

**Software:** Deniz Akbulut.

**Validation:** Deniz Akbulut.
